# Cross-modal association analysis and matching model construction of perceptual attributes of multiple colors and combined tones

**DOI:** 10.3389/fpsyg.2022.970219

**Published:** 2022-12-06

**Authors:** Shuang Wang, Jingyu Liu, Xuedan Lan, Qihang Hu, Jian Jiang, Jingjing Zhang

**Affiliations:** ^1^State Key Laboratory of Media Convergence and Communication, Communication University of China, Beijing, China; ^2^Key Laboratory of Acoustic Visual Technology and Intelligent Control System, Ministry of Culture and Tourism, Communication University of China, Beijing, China; ^3^Beijing Key Laboratory of Modern Entertainment Technology, Communication University of China, Beijing, China; ^4^China Digital Culture Group Co., Ltd, Beijing, China; ^5^School of Computer and Cyber Sciences, Communication University of China, Beijing, China

**Keywords:** multiple colors, combined tones, multi-color perception, interval consonance, audio-visual cross-modal matching model, subjective evaluation experiment, correlation analysis, machine learning

## Abstract

Audio-visual correlation is a common phenomenon in real life. In this article, aiming at analyzing the correlation between multiple colors and combined tones, we comprehensively used experimental methods and technologies such as experimental psychology methods, audio-visual information processing technology, and machine learning algorithms to study the correlation mechanism between the multi-color perceptual attributes and the interval consonance attribute of musical sounds, so as to construct an audio-visual cross-modal matching models. Specifically, in the first, this article constructed the multi-color perceptual attribute dataset through the subjective evaluation experiment, namely “cold/warm,” “soft/hard,” “transparent/turbid,” “far/near,” “weak/strong,” pleasure, arousal, and dominance; and constructed the interval consonance attribute dataset based on calculating the audio objective parameters. Secondly, a subjective evaluation experiment of cross-modal matching was designed and carried out for analyzing the audio-visual correlation, so as to obtain the cross-modal matched and mismatched data between the audio-visual perceptual attributes. On this basis, through visual processing and correlation analysis of the matched and mismatched data, this article proved that there is a certain correlation between multicolor and combined tones from the perspective of perceptual attributes. Finally, this article used linear and non-linear machine learning algorithms to construct audio-visual cross-modal matching models, so as to realize the mutual prediction between the audio-visual perceptual attributes, and the highest prediction accuracy is up to 79.1%. The contributions of our research are: (1) The cross-modal matched and mismatched dataset can provide basic data support for audio-visual cross-modal research; (2) The constructed audio-visual cross-modal matching models can provide a theoretical basis for audio-visual interaction technology; (3) In addition, the research method of audio-visual cross-modal matching proposed in this article can provide new research ideas for related research.

## Introduction

Synesthesia (Grossenbacher and Lovelace, 2001; Ward, 2013) refers to the mental activity in which stimulation from one sense induces the sensation of another sense. People’s cognition of the world comes from a variety of sensory information, such as vision, hearing, smell, taste, touch, etc. The integration of multiple sensory channels helps us to have a more comprehensive understanding of things and reduces our dependence on a certain sensory channel (Jia et al., 2018). Audio-visual synesthesia belongs to one of them. For example, when listening to music, different pictures often emerge in our mind along with the music, and color is the most intuitive description of pictures and emotions. And when we appreciate a painting, there seems to be a piece of music in our ears. This cross-modal association is automatic, involuntary, and irrepressible (Li, 2021). At present, a large number of researches have been conducted on the phenomenon, process, and mechanism of the multi-sensory information integration in neurobiology, brain science, psychology, and other related fields, which results in the proposal of a series of theoretical hypotheses and models about synesthesia. The multi-sensory information integration has been scientifically verified in many fields, proving that the phenomenon of synesthesia is universal and stable, and thus can be studied (Rouw and Scholte, 2007; Kim et al., 2013; Arturo and James, 2016).

The connection between music and color studied in this article is an audio-visual synesthesia phenomenon. Music and painting are two art forms that are closely related to our lives, and the relationship between music and color is the closest. At present, there have been many studies on audio-visual synesthesia, which exploring the basic attributes of the single tone (including length of sound, pitch, loudness, etc.) and the basic attributes of the single color (including hue, chroma, lightness, etc.). In the study of the Positron Emission Tomography (PET), Bushara et al. (2001) asked subjects to determine whether pure auditory tones and color rings presented in visual form were presented at the same time, and found that the prefrontal and posterior parietal lobes of the brain are the part of network of multi-sensory brain regions that detects the synchronization of visual and auditory stimuli. Eimer and Schröger (2003) used the functional Magnetic Resonance Imaging (fMRI) to investigate the neurophysiological mechanism of audio-visual cross-modal integration of speech signals, and found that there is a significant integration effect in the posterior part of the left superior temporal sulcus. In addition, the psychological phenomenon of Mcgurk and Macdonald (1976) shows that the process of forming cognition of external information is a process of forming a holistic understanding of things based on different sensory information. The lack or inaccuracy of any sensory information will lead to the brain’s comprehension deviation of external information. Under certain circumstances, the sound obtained simply relying on the ear is different from the sound obtained by combining visual and auditory sensory information. This phenomenon provides support for cross-modal interaction among people’s various senses, and is also a strong evidence of audio-visual synesthesia. Therefore, many studies have shown that the audio-visual cross-modal integration effect is universal and stable for people with different ages, genders, and cultural backgrounds.

In recent years, the research on audio-visual cross-modal has gradually become a hotspot. Zhou (2004) used the method of experimental psychology, took the synesthesia as the breakthrough point, and proved that there is a certain relationship between visual attributes and auditory attributes through qualitative research. Palmer et al. (2013) demonstrated that the cross-modal matches between music and colors are mediated by emotional associations. In the field of information science, Jiang et al. (2019) put forward the method and idea of cross-modal research on audio-visual integration effect, which regarded the human brain as a “black box” and divided the “input” of visual and auditory information into low-level features, middle-level features, and high-level features. The low-level features refer to objective physical features, including visual color, shape, texture, etc., and auditory pitch, loudness, etc. Due to the different data representation of different modalities, the low-level features are quite different, so direct audio-visual association cannot be carried out at this layer. The middle-level features refer to perceptual features, including visual perceptual features (e.g., color’s “warm/cool,” “swell/shrink,” “dynamic/static,” and the harmony of color combination, etc.) and auditory perceptual features (e.g., fullness, roughness, and the interval consonance of musical sounds). Perceptual features refer to physiological reflexes directly generated by physical stimuli, and there are certain similarities among different modalities. High-level features refer to semantic features, including emotion, aesthetic feeling, etc. Semantic features are the results of integrating different-modal information received by human brain, and different-modal information is shared at this layer. On the basis of this, Liu et al. (2021) extracted timbre perceptual features and quantitatively analyzed them with the basic attributes of single color (hue, chroma, and lightness) to construct the timbre-color cross-modal matching model. The research results showed that certain attributes of timbre have a strong correlation with certain attributes of color.

Most of the current studies focus on the cross-modal correlation between single color and single tone. However, in real life, pictures are composed of multiple colors, and music is composed of combined tones, which are more common and applicable. Moreover, studies have shown that there are differences in perceptual attributes between single color and multiple colors, single tone and combined tones (Griscom and Palmer, 2012). Therefore, it is necessary to further study the cross-modal association between multiple colors and combined tones on the basis of studying the correlation between single color and single tone, so as to enrich the theoretical and methodological research of audio-visual synesthesia and reveals the essence of synesthesia. Among them, the classic color matching mode of multiple colors is three-color combination (Kato, 2010). Therefore, this article took three-color combinations as the visual materials and quantified the corresponding perceptual attributes. On the other hand, the basic unit of combined tones is the interval, which refers to the mutual relationship between two tones in pitch (including harmonic interval and melodic interval), and the interval consonance is the basic perceptual attribute reflecting the auditory perception of the interval (Wang and Meng, 2013; Lu and Meng, 2016). Therefore, this article took intervals as the auditory materials and quantified the interval concordance attribute.

**FIGURE 1:**
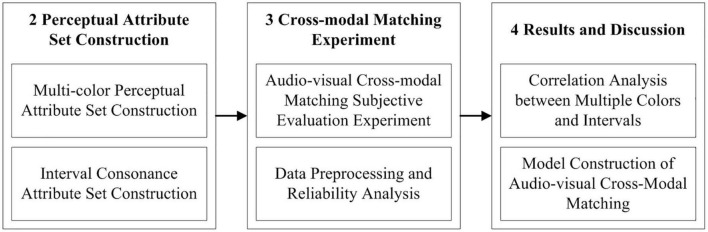
The section arrangement of this article.

To sum up, this article took multiple colors and combined tones as the research object, designed and implemented the audio-visual cross-modal matching subjective evaluation experiment between three-color combinations and intervals, analyzed the audio-visual cross-modal correlation on the basis of quantifying the relevant perceptual attributes of audio-visual materials, and finally constructed the audio-visual cross-modal matching models through the linear and non-linear machine learning algorithms. The section arrangement of this article is shown in Figure 1.

## Perceptual attribute dataset construction

This section introduces the construction of multi-color perceptual attribute dataset and interval consonance attribute dataset, mainly including the construction of audio-visual material sets and the quantification of audio-visual perceptual attributes, which provided data supporting for the research on the association between multi-color perception and interval consonance.

### Multi-color perceptual attribute dataset construction

#### Multi-color material set construction

Fifty three-color combinations selected from the research results of Nippon Color & Design Research Institute (NCD) were used as the multi-color materials. NCD selected 130 representative colors which represented psychological experiences accurately based on the “Hue and Tone System,” which were evaluated by 180 image descriptive words. Then, they combined 130 single color into 50 three-color combinations based on color image and further classified them into a total of 16 categories, including “lovely,” “romantic,” and “refreshing,” as shown in Supplementary Appendix Table A1. On this basis, cluster analysis was adopted to reduce dimensions of 180 image descriptive words, so as to construct a three-dimensional “color image scale,” namely the dimension of “cool/warm,” “soft/hard,” and “transparent/turbid,” and mapped 16 categories into this color image space (Kobayashi, 2006, 2010). These 50 materials and their values of L*, a*, and b* in the CIELAB color space are respectively shown in Supplementary Appendix Table A2.

#### Subjective evaluation experiment on multi-color perception

The multi-color perceptual attributes refer to the quantitative description of three-color combinations from human’s perception, such as the subjective senses of “cool/warm,” “far/near,” and “pleasure” inspired by color. In this article, the data of multi-color perceptual attributes was obtained through the subjective evaluation experiment on color perception. Then, the experimental data would be applied into the correlation analysis between multi-color perceptual attributes and interval consonance attribute, and the construction of audio-visual cross-modal matching models.

##### Subjects

A total of 16 subjects, aged 18–22, with the gender ratio close to 1:1, took part in the experiment. None of them majored in visual science or aesthetic design. Before the formal experiment, each subject was given an Ishihara Color Blindness Test (Marey et al., 2015) to ensure his color vision was normal. Then, they would sign the informed-consent form and were compensated for participating.

##### Perceptual descriptive words selection

A total of 260 perceptual descriptive words were selected from literature, questionnaires, and dictionaries (Gao and Xin, 2006; Wang et al., 2006, 2020), and then they were further screened according to the following principles: (1) remove words which are susceptible to individual preference; (2) combine words with similar meanings; (3) remove words with ambiguous explanations. On this basis, a preliminary experiment (Kobayashi, 2010) was carried out to select perceptual descriptive words based on multi-color materials, and the subjects were asked to select several words which were suitable for describing a certain three-color combination. The selection frequency of each word was recorded to determine the selected one. The selected perceptual descriptive words could not be affected by individual preference and had objectivity and universality. Finally, as shown in Table 1, five pairs of mid-level descriptive words were selected, namely “cool/warm,” “soft/hard,” “transparent/turbid,” “far/near,” and “weak/strong.” In addition, the remaining three high-level descriptive words were from the PAD emotion model with *P* representing pleasure, *A* representing arousal, and *D* representing dominance (Russell and Mehrabian, 1977), so as to acquire the more complete description for human’s multi-color perception.

**TABLE 1:** The selected perceptual descriptive words of multi-color perceptual attributes.

No.	Chinese descriptive word	English name, abbreviation	Description
1	冷/暖	Cool/warm, CW	• Cool: (feel) lower temperature; • Warm: (feel) higher temperature.
2	软/硬	Soft/hard, SH	• Soft: loose internal structure, easy to deform; • Hard: tight internal structure, difficult to deform.
3	透明/浑浊	Transparent/turbid, TT	• Transparent: (an object) can penetrate light; • Turbid: contain impurities.
4	远/近	Far/near, FN	• Far: The distance in space is long. • Near: The distance in space is short.
5	弱/强	Weak/strong, WS	• Weak: Small in number and shallow in degree. • Strong: The degree is very high.
6	愉悦度	Pleasure, P	The positive and negative characteristics of an individual’s emotional state, including the two opposing states of positive or negative emotion.
7	激活度	Arousal, A	The individual’s neurophysiological activation level, which is related to the activation degree of the body’s energy associated with emotional states, including two states of low arousal (such as quiet) and high arousal (such as excited).
8	优势度	Dominance, D	The individual’s state of control over the situation and others, and is used to distinguish whether the emotional state is subjectively issued by the individual or the influence of the objective environment, including active and passive states.

“Cool/warm,” “soft/hard,” “transparent/turbid,” “far/near,” and “weak/strong” belong to the mid-level perceptual descriptive words, and pleasure, arousal, and dominance belong to the high-level perceptual descriptive words.

##### Experimental condition

The experiment was carried out in an underground standard listening room with a reverberation time of 0.3 s. The sound field distribution was uniform, and there was no bad acoustic phenomenon and body noise, so as to avoid the interference of noise on the perceptual evaluation. The laboratory area was 5.37 m × 6 m, and the wall acoustic absorption materials and the main experimental facilities were all gray (Munsell: N4). A 75-inch Sony KD-75X9400D HD display was adopted with a resolution of 4K (3840 2160). According to the Methodology for the Subjective Assessment of the Quality of Television Pictures (ITU-R BT.500-14), the ambient illumination of the display was set to 200 lx. A “slideshow” function in the ACDSee software (official free version; ACD Systems International Inc., Shanghai, China) was used to randomly present the stimuli, which was centered with a gray background (Munsell: N2). The luminance is 596.5 cd/m2, and the luminance uniformity is 0.03 cd/m2 (Input signal: black level; Value: SD; Method: Nine-point Test) and 10.69 cd/m2 (Input signal: white level; Value: SD; Method: Nine-point Test).

##### Experimental procedure

The experiment was divided into two steps. The first step was to fill in the basic personal information and sign the informed-consent form. The second step was to evaluate the perceptual descriptive words on a five-level scale. In the experiment, the scoring time of each material was uniformly controlled for 30 s, and the interval between two pictures was 5 s. In order to avoid eye fatigue caused by long-term viewing of the screen, the materials were divided into two groups. After 25 materials were evaluated, a 5-min break was taken before the remaining 25 materials were evaluated.

#### Reliability analysis and multi-color perceptual attributes quantification

Cronbach’s alpha was adopted to evaluate the reliability of the experimental data, which is used to measure the internal consistency of the evaluation results, usually above 0.7 is considered to be reliable.


(1) $$\alpha =\frac{k}{k-1}\,\left(1-\frac{\sum_{i=1}^{k}\sigma_{i}^{2}}{\sigma_{x}^{2}}\right)$$

where *K* represents the number of subjects, $\sigma_{i}^{2}$ repreents the score variance of all the subjects on the *ith* measurement item, and $\sigma_{x}^{2}$ represents the total variance of the total scores obtained by all the subjects. All the perceptual descriptive words have very good internal consistency among the 16 subjects, and the Cronbach’s alpha are: 0.94 (“cool/warm”), 0.908 (“soft/hard”), 0.946 (“transparent/turbid”), 0.715 (“far/near”), 0.921 (“weak/strong”), 0.893 (pleasure), 0.917 (arousal), and 0.875 (dominance). It can be seen that the values of the Cronbach’s alpha are all greater than 0.7, and the highest value is up to 0.946, which meets the reliability requirements.

Finally, as shown in Equation (2), an eight-dimensional multi-color perceptual attribute vector was constructed based on the evaluation results.


(2) F=[f,1f,2…,f]8

where *f_i_* represents the average value of 5-scale scores of all the subjects on the *ith* multi-color perceptual descriptive word.

### Interval consonance attribute dataset construction

#### Interval material set construction

**FIGURE 2:**
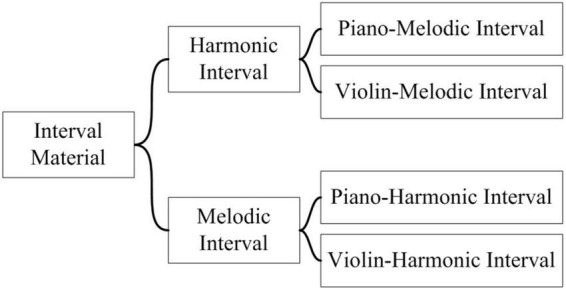
The composition of the interval materials.

The composition of the interval materials is shown in Figure 2, with a total of 52 interval materials, including melodic intervals and harmonic intervals. Among them, a melodic interval refers to a two-tone combination played successively, and a harmonic interval refers to a two-tone combination played synchronously. Each interval category composes of two timbres, namely piano (unsustainable sound) and violin (sustainable sound). A musical instrument includes four phase: Attack phase, Decay phase, Sustain phase, and Release phase, which are called ADSR model for short. “unsustainable sound” means that there is only a simplified ADSR model for musical instruments, and only the attack phase and decay phase, such as plucked instruments (harps), percussion instruments (pianos), and percussion instruments (marimba). “Sustainable sound” refers to the sustain phase of musical instruments in the ADSR stage, such as violin, clarinet and trumpet (Jiang et al., 2020). In general, there are 13 two-tone relationships with different interval consonance indexes, as shown in Table 2. Therefore, there are a total of four categories, namely piano-melodic interval, violin-melodic interval, piano-harmonic interval, and violin-harmonic interval, with 13 intervals respectively.

**TABLE 2:** The 13 common two-tone relationships (intervals).

No.	Intervals	Abbreviation
1	Perfect unison	P1
2	Minor second	m2
3	Major second	M2
4	Minor third	m3
5	Major third	M3
6	Perfect forth	P4
7	Augmented forth	A4
8	Perfect fifth	P5
9	Minor sixth	m6
10	Major sixth	M6
11	Minor seventh	m7
12	Major seventh	M7
13	Octave	P8

The MacBook Pro personal computer and the Sony 8506 monitor earphone were adopted to record interval materials. The steps were as follows: (1) open the digital audio workstation software Logic Pro (X; Apple Inc., Cupertino, CA, USA), and create the new project file; (2) establish two audio tracks of two kinds of instruments, namely piano and violin. Taking the M3 harmonic interval played by piano as an example, import the Kontakt6 sampler into these two audio tracks, and add the piano sound source (Cine Piano, set to the default value) and the violin sound source (Chris Hein Solo Violin, set to Dynamic Expression Long); (3) write the melodic intervals and harmonic intervals with the loudness of mezzo forte (mf) in two audio tracks respectively, and export all the interval materials.

#### Interval consonance attribute quantification

Based on the research results of the previous literatures (Chen and Lu, 1994; Xue, 2012), according to the harmony principle, a quantitative research method was given for calculating the interval consonance index of the pure-tone and 12-tone equal temperament, and classified the 13 intervals into six categories based on the interval consonance index. The principle of the calculation of interval consonance index is: if the two tones have the same partial, that is, the frequency ratio of the two tones is a simple integer ratio, then the effect of consonance can be produced. The calculation steps are as follows.

**Step 1**: Calculate the consonance degree of each pure-tone interval firstly. Take the reciprocal of the product of the ordinal numbers of the first consonant partials of the two tones constituting the interval in the respective partial columns as the basis for evaluating the consonance degree, which is called the interval consonance coefficient *K*:


(3) $$K=\frac{1}{m\,n}$$

where *m* represents the ordinal number of the first consonant partial in the root-tone partial column and *n* represents the ordinal number of the first consonant partial in the crown-tone partial column. The partial column of the tones in the pure-tone temperament is shown in Table 3. It can be seen that the tones with different frequency can have the same partial, which is called the consonant partial. Take the interval composed of g and c (P5) as the example. The first consonant partial is g^1^, which is the second partial in the g partial column and the third partial in the c partial column. Therefore, the interval consonance coefficient $K=\frac{1}{2\times 3}=\frac{1}{6}$.

**TABLE 3:** The partial column of the tones in the pure-tone temperament relative to c (from the first partial to the 16th partial).

Tone name	c	d	e	f	g	a	b	c^1^
								
Frequency ratio	1/1	9/8	5/4	4/3	3/2	5/3	15/8	2/1
								
Cent value	0	204	386	498	702	884	1088	1200
The first partial	c	d	e	f	g	a	b	c^1^
The second partial	c^1^	d^1^	e^1^	f^1^	g^1^	a^1^	b^1^	c^2^
The third partial	g^1^	a^1^	b^1^	c^2^	d^2^	e^2^	f^2^	g^2^
The 4th partial	c^2^	d^2^	e^2^	f^2^	g^2^	a^2^	b^2^	c^3^
The 5th partial	e^2^	f^2^	g^2^	a^2^	b^2^	c^3^	d^3^	e^3^
The 6th partial	g^2^	a^2^	b^2^	c^3^	d^3^	e^3^	f^3^	g^3^
The 7th partial	969	1173	155	267	471	653	857	969
The 8th partial	c^3^	d^3^	e^3^	f^3^	g^3^	a^3^	b^3^	c^4^
The 9th partial	d^3^	e^3^	f^3^	g^3^	a^3^	b^3^	c^4^	d^4^
The 10th partial	e^3^	f^3^	g^3^	a^3^	b^3^	c^4^	d^4^	e^4^
The 11th partial	551	755	937	1049	53	235	439	551
The 12th partial	g^3^	a^3^	b^3^	c^4^	d^4^	e^4^	f^4^	g^4^
The 13th partial	841	1045	27	139	343	525	729	841
The 14th partial	969	1173	155	267	471	653	857	969
The 15th partial	b^3^	c^4^	d^4^	e^4^	f^4^	g^4^	a^4^	b^4^
The 16th partial	c^4^	d^4^	e^4^	f^4^	g^4^	a^4^	b^4^	c^5^

Only partial partials under the 16th cent are listed. Among them, the pitch is indicated by the name of the pure tone, and theserial number of the partial column is indicated by the superscript numbers.

**Step 2**: Since the distribution of *K* is not uniform, the logarithmic method was adopted to define the interval consonance index of the pure-tone temperament *Ip*. And the unit is decibel (dB).


(4) $$I\,p=20\,l\,o\,g_{10}\,\frac{1000}{m\,n}\,\text{dB}$$

**Step 3**: Since the frequency ratio of each interval of the twelve-tone equal temperament currently in use is an irrational number ${\left(\sqrt[{12}]{2}\right)}^{k}$, where *k* = 1,2,⋯,12, the calculation method of the interval consonance index of the pure-tone temperament is not suitable for that of the 12-tone temperament. In practice, the similar interval of the pure-tone temperament is commonly adopted to calculate the interval consonance index *I* of the corresponding twelve-tone equal temperament:


(5) I=I⁢p-△⁢I

where *I* represents the interval consonance index of the similar interval of the pure-tone temperament; −△*I* represents the correction value corresponding to the deviated cent δ of the twelve-tone equal temperament interval from the similar pure-tone temperament interval. −△*I* is calculated as follows:


(6) $$l\,o\,g\,\eta =\frac{\delta \,l\,o\,g\,2}{1200}$$

(7) $$A=\frac{1}{\sqrt{1+Q^{2}\,{\left(\eta -\frac{1}{\eta }\right)}^{2}}}$$

(8) $$-△\,I=20\,l\,o\,g_{10}\,A$$

where δ represents the cent value of a certain interval deviating from a similar one of the pure-tone temperament; η represents the frequency ratio corresponding to δ; *A* represents the relative amplitude; *Q* represents the quality factor of the resonant circuit, which is set to 100. The calculation results are shown in Table 4.

**TABLE 4:** The interval consonance index of each interval of the 12-tone equal temperament (*Q* = 100).

	12-Tone equal temperament		Similar pure- temperament		δ	−△I (dB)	I (dB)	Category
						
No.	Interval	Cent	Cent	I*p* (dB)				
1	P1	0	0	60	0	0	60	A
2	m2	100	112	12.4	12	–4.5	7.9	E
3	M2	200	204	20.85	4	–0.9	19.95	D
4	m3	300	316	30.46	16	–6.3	24.16	C
5	M3	400	386	33.98	14	–5.4	28.58	C
6	P4	500	498	38.42	2	–0.22	38.22	B
7	A4	600	610	29.19	10	–3.6	25.59	C
8	P5	700	702	44.44	2	–0.22	44.22	B
9	m6	800	814	27.96	14	–5.4	25.56	C
10	M6	900	884	36.48	16	–6.3	30.18	C
11	m7	1000	996	16.83	4	–0.9	15.93	D
12	M7	1100	1088	18.42	12	–4.5	13.92	E
13	P8	1200	1200	53.98	0	0	53.98	A

A represents “very complete consonance,” B represents “complete consonance,” C represents “incomplete consonance,” D represents “incomplete consonance,” and E represents “very incomplete consonance.”

## Audio-visual cross-modal matching subjective evaluation experiment

Firstly, on the basis of research results of Section “Perceptual Attribute Dataset Construction,” this section introduces the design and implementation process of the audio-visual cross-modal matching subjective evaluation experiment between audio-visual perceptual attributes. Then, data preprocessing and reliability analysis were introduced, which provided the input and output data for correlation analysis and model construction.

The experiment was carried out by selecting the corresponding multi-color materials after listening to the interval materials. In the experiment, the independent variable was the playing interval material, and the dependent variable was the corresponding matched or mismatched multi-color material.

### Subjects

A total of 23 subjects participated in the experiment, including 7 males and 16 females, all aged between 18 and 22 years old, and their majors were not related to audio-visual science. Before the experiment, each subject was given the Ishihara Color Blindness Test to ensure their color vision normal. Then, everyone signed an informed-consent form and was compensated for their participation. In addition, the subjects were different from that of the multi-color perception experiment carried out before.

### Experimental condition

**FIGURE 3:**
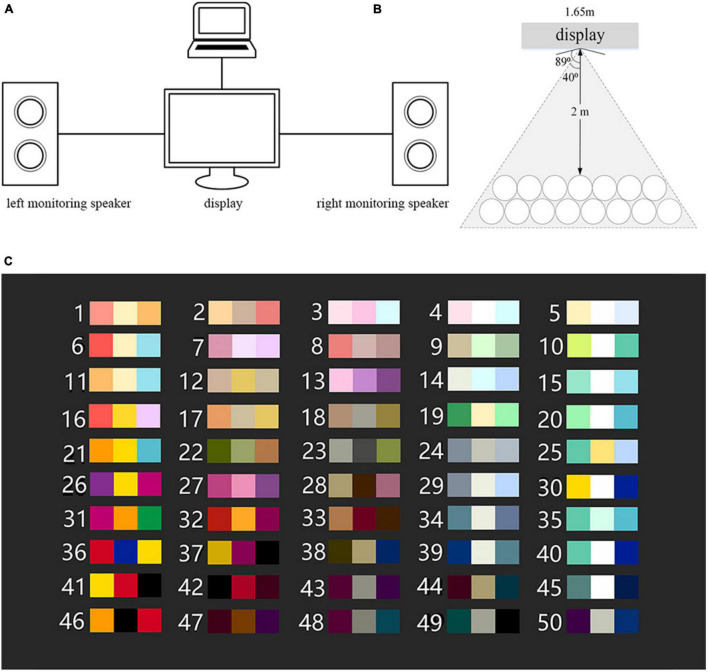
The experimental condition. **(A)** The connection of the experimental system. **(B)** The arrangement of seats. **(C)** The presentation of the multi-color materials.

The experiment was carried out in the standard listening room. The experimental condition of sound field and visual material presentation were the same as that of the multi-color perception experiment (see section “Subjective evaluation experiment on multi-color perception”). The three-way midfield monitoring speaker Genelec 1038B was used to broadcast the interval materials. In the whole experiment process, the sound pressure level of the sound signal remained unchanged, and the actual sound pressure level was 75 dB (A), in line with the Standard of Acoustics Measurement in Hall (GB 50371-2006). The connection of the experimental system is shown in Figure 3A. One end of the laptop was connected to the display to realize the presentation of the multi-color materials, and the other end was connected to the left monitoring speaker and the right speaker respectively to realize the play of interval materials. The Adobe Audition software (14.1 version; Adobe Systems Incorporated, San Jose, CA, USA) was used to play interval materials. During the experiment, the ear height of the subjects should be at the same level as the midpoint of the vertical line in the high and low sounds of speakers.

In addition, as shown in Figure 3B, the seats were arranged in an arc with the monitor as the center, so as to make the distance between the subjects and the monitor fixed at 2 m. The horizontal viewing angle of the display is 178° which is the maximum angel at which an user can clearly see the screen, and the actual viewing angle from the subjects sitting on either of two sides is 80, so as to ensure the consistency of color displaying. The presentation of the multi-color materials is shown in Figure 3C, with a gray background (Munsell: N2).

### Experimental procedure

The experiment was conducted in two groups with 11 or 12 subjects in each group. During the experiment, the subjects were prohibited to talk or signal to each other. The experiment was divided into three stages, namely familiarization stage, training stage, and formal experiment stage, which is shown below.

**(1) Familiarization stage**: The interval materials were played in order, so as to avoid excessive concentration of subjective scores during the formal experiment.

**(2) Training stage**: Three interval materials were randomly selected from the material set, and the subjects were asked to select corresponding multi-color materials according to their subjective feelings, so as to avoid the influence caused by unfamiliarity with the experimental procedure.

**(3) Formal experiment stage**: Play each interval material randomly, and the subjects were asked to select the corresponding first matched, the second matched, and the third matched multi-color materials. Similarly, the subjects were then asked to select the first mismatched, the second mismatched, and the third mismatched multi-color materials.

In order to ensure the accuracy of the results, the subjects were asked to make the immediate choices based on their subjective feelings, and the playing time of each interval material was limited to 30 s. On the other hand, 64 interval materials (including 12 repeated interval materials selected randomly to verify the test–retest reliability) were divided equally into four groups, with a 5-min break between each group, so as to avoid the fatigue effect.

### Data preprocessing and reliability analysis

**FIGURE 4:**
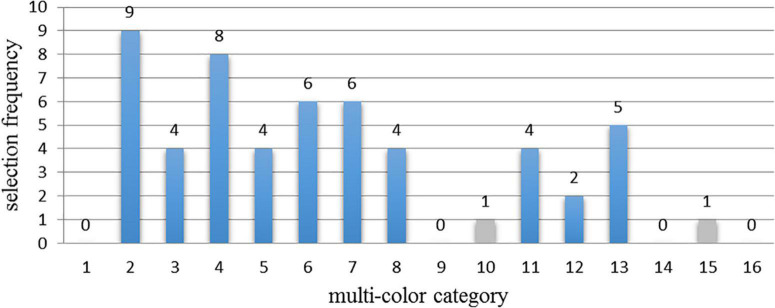
The example of the histogram of the selection frequency of 16 multi-color categories which match the interval material 1 (P1 piano-harmonic interval). Among them, the gray bar represents the removed data in the histogram.

Firstly, observing the experimental data, it can be seen that each set of matching data had multi-color materials that were selected only once. Therefore, in order to avoid the influence of abnormal data on the experimental results, for each set of the experimental data, all the data corresponding to the categories with the least selection frequency were removed. Taking the histogram of the P1 piano-harmonic interval as the example, as shown in Figure 4, the selection frequency of category 10 and 15 of multi-color materials was only once. Therefore, the data from category 10 and category 15 were supposed to be removed.

Then, the Cronbach’s alpha was adopted to evaluate the reliability of the experimental data, and the Cronbach’s alpha are: 0.861 (piano-harmonic intervals), 0.795 (piano-melodic intervals), 0.874 (violin-harmonic intervals), and 0.716 (violin-melodic intervals) respectively, which meets the reliability requirements.

Finally, the linearly weighted average values (Palmer et al., 2013) of the multi-color perceptual attributes of the matched and mismatched materials were used as the values of the multi-color perceptual attributes. The calculation methods are shown in Equation (9) and (10).


(9) $$C_{d,m}=\left(3\times C_{1,d,m}+2\times C_{2,d,m}+C_{3,d,m}\right)/6$$

(10) $$I_{d,m}=\left(3\times I_{1,d,m}+2\times I_{2,d,m}+I_{3,d,m}\right)/6$$

where *C*_*j,d,m*_ represents the value of the multi-color perceptual attribute *d* of the *jth* matched multi-color material for the *mth* interval material selected by the subjects. For example, *C*_*1,cool/warm,1*_ represents the “cool/warm” value of the first matched multi-color material selected by the subjects for the P1 piano-harmonic interval. *I*_*j,d,m*_ represents the value of the perceptual attribute *d* of the *jth* mismatched multi-color material for the *mth* interval material selected by the subjects. For example, *I*_*2,soft/hard,2*_ represents the “soft/hard” value of the second mismatched multi-color material for the m2 piano-harmonic interval. In addition, *j* ∈ [1,3], and *m* ∈ [1,52].

## Results and discussion

This section conducted the correlation analysis of the matched and mismatched relationships between the perceptual attributes of multiple colors and intervals, including drawing scatterplots and calculating correlation coefficients. Then, the linear and non-linear machine learning algorithms were adopted to construct the audio-visual cross-modal matching models between multiple colors and intervals, and further analyzed the relationship between these two modes.

### Correlation analysis between multiple colors and intervals

After obtaining the correlation data of multi-color perceptual attributes and interval consonance attribute through the subjective matching experiment, it was necessary to draw scatterplots and fitting curves to visually analyze the correlation with abnormal distribution removed. Then, the Pearson’s correlation coefficient was calculated for the quantitative analysis.

### Scatterplot analysis

**FIGURE 5:**
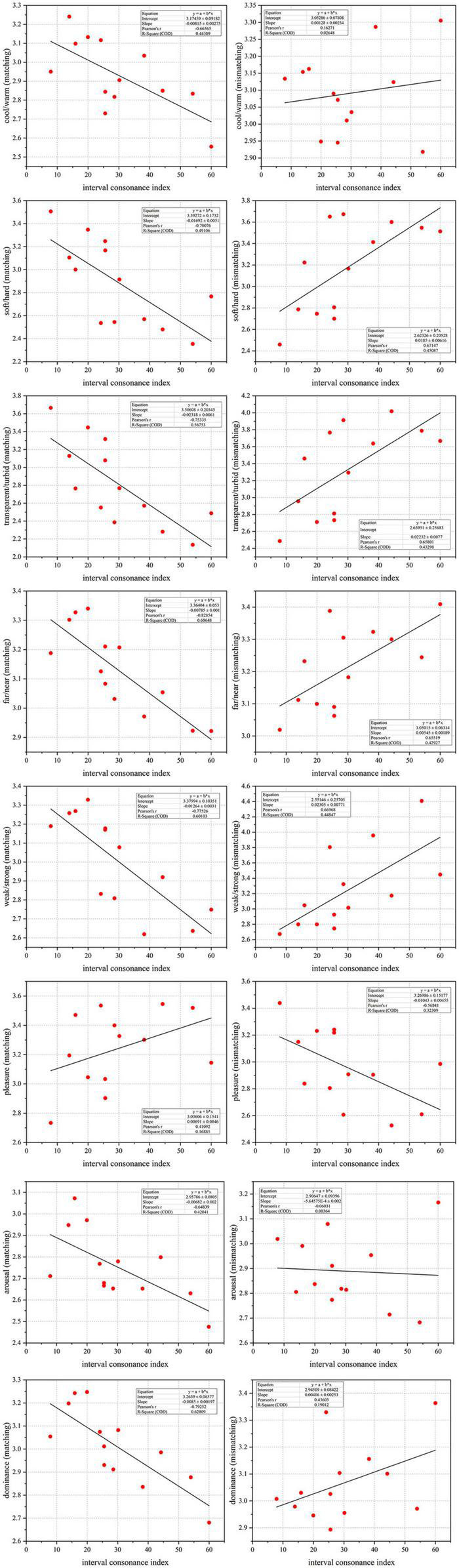
The scatterplots between audio-visual perceptual attributes. Among them, the *X*-axis represents the interval consonance index (*I*) and the *Y*-axis represents a certain perceptual attribute of matched (or mismatched) multi-color material corresponding to the interval material.

In order to study the correlation between multiple colors and intervals, the scatterplot was drawn according to the interval consonance index (*I*), the perceptual attributes of multiple colors and the matched and mismatched relationships between them in the first, as shown in Figure 5. The *X*-axis represents the interval consonance index (*I*) and the *Y*-axis represents a certain perceptual attribute of matched (or mismatched) multi-color material corresponding to the interval material. Due to the large number of scatter plots, only the significant linear matched and mismatched relationship between the multi-color perceptual attributes and the piano-harmonic interval consonance index (*I*) are shown below.

As can be seen from Figure 5, there is a significant correlation between some multi-color perceptual attributes and the interval consonance attribute, which is opposite between the matched and mismatched relationship. For example, the more consonant piano-harmonic interval corresponds to the cooler, softer, more transparent, further, weaker, less arousable, and less dominant matched multi-color perception. On the contrary, the corresponding mismatched multi-color perception is harder, more turbid, nearest, stronger, and less pleasant.

### Correlation coefficient analysis

To further analyze the relationship between multi-color perceptual attributes and the interval consonance attribute, the Pearson’s correlation coefficient (*r*) was adopted to analyze the correlation between the interval consonance attribute and its corresponding matched (or mismatched) multi-color perceptual attributes. If |*r*|≥0.8, there is a strong correlation; if 0.5≤|*r*| < 0.8, there is a medium correlation; if 0.3≤|*r*| < 0.5, there is a weak correlation; if |*r*| < 0.3, there is no correlation. The calculation results of the Pearson’s correlation coefficient are shown in Table 5.

**TABLE 5:** The Pearson’s correlation coefficient matrix between multi-color perceptual attributes and the interval consonance attribute.

	Piano-harmonic intervals	Piano-melodic intervals	Violin-harmonic intervals	Violin-melodic intervals
Cool/warm (M)	–0.666	0.069	0.221	–0.342
Cool/warm (N)	0.163	0.449	–0.194	0.234
Soft/hard (M)	–0.701	–0.626	–0.259	–0.34
Soft/hard (N)	0.671	0.395	0.244	0.477
Transparent/turbid (M)	–0.753	–0.457	–0.127	–0.522
Transparent/turbid (N)	0.658	0.308	0.085	0.428
Far/near (M)	–0.829	–0.385	–0.014	–0.173
Far/near (N)	0.655	0.641	–0.088	0.189
Weak/strong (M)	–0.775	–0.674	–0.228	–0.155
Weak/strong (N)	0.67	–0.019	0.29	0.096
Pleasure (M)	0.411	0.167	0.164	0.246
Pleasure (N)	–0.568	0.024	–0.144	–0.2
Arousal (M)	–0.648	–0.268	0.077	–0.067
Arousal (N)	0.06	0.317	0.049	0.142
Dominance (M)	–0.793	–0.281	–0.009	–0.023
Dominance (N)	0.436	0.497	0.147	0.189

M represents “match,” and N represents “no match.” Then, represents the strong correlation, represents the medium correlation, and represents the weak correlation.

It can be seen from Table 5, some multi-color perceptual attributes are significantly correlated with the interval consonance attribute. First of all, under the matched relationship between the multi-color perceptual attributes and the piano-harmonic interval consonance attribute, there is a strong correlation between the “far/near” multi-color perceptual attribute and the piano-harmonic interval consonance attribute, with the highest Pearson’s correlation coefficient: *r* = −0.829,*p* = 0.001. Then, there is a medium correlation between the multi-color perceptual attributes, namely “cool/warm,” “soft/hard,” “transparent/turbid,” “weak/strong,” arousal, and dominance, and the piano-harmonic interval consonance attribute, with the Pearson’s correlation coefficients are: *r* = −0.666,*p* = 0.001, *r* = −0.701,*p* = 0.001, *r* = −0.753,*p* = 0.001, *r* = −0.775,*p* = 0.001, *r* = −0.648,*p* = 0.001, and *r* = −0.793,*p* = 0.001 respectively. And there is a weak correlation between the pleasure multi-color perceptual attribute and the piano-harmonic interval consonance attribute, with the Pearson’s correlation coefficient: *r* = 0.411,*p* = 0.001. Under the matched relationship between the multi-color perceptual attributes and the piano-melodic interval consonance attribute, there is a medium correlation between the multi-color perceptual attributes, namely “soft/hard” and “weak/strong,” and the piano-melodic interval consonance attribute, with the Pearson’s correlation coefficients are: *r* = −0.626,*p* = 0.001 and *r* = −0.674,*p* = 0.001. And there is a weak correlation between the multi-color perceptual attributes, namely “transparent/turbid” and “far/near,” and the piano-melodic interval consonance attribute, with the Pearson’s correlation coefficients are: *r* = −0.457,*p* = 0.001 and *r* = −0.385,*p* = 0.001. On the contrary, there is no correlation between the multi-color perceptual attributes and the violin-harmonic interval consonance attribute under the matched relationship. Then, there is a medium correlation between the “transparent/turbid” multi-color perceptual attribute and the violin-melodic interval consonance attribute, with the Pearson’s correlation coefficient is: *r* = −0.552,*p* = 0.001. And there is a weak correlation between the multi-color perceptual attributes, namely “cool/warm” and “soft/hard,” and the violin-melodic interval consonance attribute, with the Pearson’s correlation coefficients are: *r* = −0.342,*p* = 0.001 and *r* = −0.34,*p* = 0.001 respectively.

Under the mismatched relationship, there is a medium correlation between the multi-color perceptual attributes, namely “soft/hard,” “transparent/turbid,” “far/near,” “weak/strong,” and pleasure, and the piano-harmonic intervals consonance attribute, with the Pearson’s correlation coefficients are: *r* = 0.671,*p* = 0.001, *r* = 0.658,*p* = 0.001, *r* = 0.655,*p* = 0.001, *r* = 0.67,*p* = 0.001, and *r* = −0.568,*p* = 0.001. Then, there is a weak correlation between the dominance multi-color perceptual attribute and the piano-harmonic interval consonance attribute, with the Pearson’s correlation coefficient is: *r* = 0.436,*p* = 0.001. For piano-melodic intervals, the “far/near” multi-color perceptual attribute has a medium correlation with the interval consonance attribute, with the correlation coefficient is: *r* = 0.641,*p* = 0.001. In addition, there is a weak correlation between the multi-color perceptual attributes and the piano-melodic interval consonance attribute, namely “cool/warm,” “soft/hard,” “transparent/turbid,” arousal, and dominance, with the correlation coefficients are: *r* = 0.449,*p* = 0.001, *r* = 0.395,*p* = 0.001, *r* = 0.308,*p* = 0.001, *r* = 0.317,*p* = 0.001, and *r* = 0.497,*p* = 0.001. Similar to the matched relationship, there is no correlation between the multi-color perceptual attributes and the violin-harmonic interval consonance attribute. Finally, for violin-melodic intervals, there is a weak correlation between the “soft/hard,” “transparent/turbid” multi-color perceptual attributes and the violin-melodic interval consonance attribute, with the correlation coefficients are: *r* = 0.477,*p* = 0.001 and *r* = 0.428,*p* = 0.001.

To sum up, the unsustainable sound signals (piano intervals) is more correlated with multi-color perception than that of the sustainable sound signals (violin intervals). Among them, the piano-harmonic interval consonance attribute is most significantly correlated with the multi-color perceptual attributes. In addition, for piano intervals, the harmonic interval consonance attribute is more correlated with multi-color perception than that of the melodic intervals, which is in contrast to violin. On the other hand, under the matched relationship, the association between audio-visual perceptual attributes is more significant than that of the mismatched relationship. Specifically, for the multi-color perceptual attributes, “weak/strong” is most correlated with the piano interval consonance attribute under the matched relationship. And “soft/hard” is most correlated with the violin interval consonance attribute under the matched relationship.

### Audio-visual cross-modal matching model construction

Based on the research results in sections “Perceptual attribute dataset construction” and “Audio-visual cross-modal matching subjective evaluation experiment,” machine learning algorithms were adopted to construct the cross-modal matching models between the multi-color perceptual attributes and the interval consonance attribute. In this section, linear and non-linear machine learning algorithms were adopted respectively, so as to further analyze the relationship between these two modes. In addition, taking the actual application scenarios into consideration, this article only constructed the audio-visual cross-modal matching model under the matched relationship.

#### Linear model construction

The Multiple Linear Regression (MLR) algorithm (Hidalgo and Goodman, 2013) has the characteristic of strong interpretability. Therefore, this section firstly used the MLR algorithm to construct the audio-visual matching models, namely the visual perception predication model (Input: the interval consonance attribute; Output: the multi-color perceptual attributes) and the audio perception predication model (Input: the multi-color perceptual attributes; Output: the interval consonance attribute). The principle of the MLR algorithm is below.

**Step 1**: Assume the linear relationship between independent variables *X*_1_,*X*_2_,…,*X*_*p*_ and the dependent variable *y* as shown in Equation (11):


(11) $$y=\beta_{0}+\beta_{1}\,X_{1}+\ldots +\beta_{p}\,X_{p}+\varepsilon$$

where β_0_,β_1_,…,β_*p*_ represents the regression parameters; ε represents the random error which obeys the distribution ofε∼N(0,σ)2.

**Step 2**: Use the Ordinary Least Square (OLS) method to estimate the regression parameters and obtain the values of β in order to minimize the objective function *Q*(β), as shown in Equation (12):


(12) $$Q\,\left(\beta \right)=m\,i\,n\,\sum_{i=1}^{n}{||y_{i}-x_{i}\,\beta ||}^{2}$$

**Step 3**: Use the *K*-fold cross validation to optimize the regression parameters, so as to reduce the random error ε, which is suitable for small data set, and is only divided once and requires relatively a little computation. We selected the 10-fold cross validation to evaluate the accuracy of the model. The original data was divided into 10 equal parts, and 9 of which were used as the training set and the remaining 1 was used as the testing set. Then, use another previously split data to replace as a testing set and repeat the above steps until each piece of data become a testing set once. Finally, calculate the average value, run the whole data again, and produce a model, which is the actual one we need.

##### Audio perception predication model

This section mainly introduced the construction of the audio perception predication model (Input: the multi-color perceptual attributes; Output: the interval consonance attribute) by the linear algorithm.

In this article, the Pearson’s correlation coefficient (*r*), the Mean Absolute Error (MAE), and the Root Mean Squared Error (RMSE) were used to evaluate the prediction accuracy of each regression model. *r* represents the statistical correlation between the true value and the predicted value, with the range from –1 to 1. The closer the *r* is to 1, the better the predication ability is. Therefore, to make the evaluation indexes more understandable, *r* was normalized to the interval [0,1]. The calculation method of MAE is shown in the Equation (13). The closer the MAE is to 0, the more accurate the model is. The calculation method of RMSE is shown in Equation (14). The closer the RMSE is to 0, the more accurate the model is.


(13) $$M\,A\,E=\frac{1}{n}\,\sum_{i=1}^{n}\left|y_{i}-f\,\left(x_{i}\right)\right|$$

(14) $$R\,M\,S\,E=\sqrt{\frac{1}{n}\,\sum_{i=1}^{n}{\left(y_{i}-f\,\left(x_{i}\right)\right)}^{2}}$$

where *y_i_* represents the true value; *f*(*x*_*i*_) represents the predictive value and *n* represents the number of training samples.

The prediction results of the interval consonance attribute by the multi-color perceptual attributes are shown in Table 6.

**TABLE 6:** The linear modeling results of the interval consonance index predicted by the multi-color perceptual attributes.

	The evaluation index		
	
	*r*	MAE	RMSE
Piano-harmonic interval	0.745	8.486	10.541
Piano-melodic interval	0.546	9.089	12.718
Violin-harmonic interval	0.115	14.907	17.499
Violin-melodic interval	0.081	21.125	26.278

It can be seen that, the predication performance of the piano-harmonic interval consonance index is good, with the evaluation indexes are: *r* = 0.745,*MAE* = 8.486,*RMSE* = 10.541. In addition, the predication performance of the piano-melodic interval consonance attribute is not very good, with the evaluation indexes are: *r* = 0.546,*MAE* = 9.089,*RMSE* = 12.718. However, others are bad, especially for the violin-melodic intervals. The prediction models of piano interval consonance attributes are shown in Equation (15) and (16).


(15) $$C\,I_{p\,\_\,h\,i}=-25.944\times C\,W+13.918\times S\,H-25.791$$

×TT-43.541×FN-12.917×

WS-15.421×P+53.862×A-27.861×D+298.87

(16) $$CI_{p\,\_\,m\,i}=-68.951\times CW-78.879\times SH+4.823\times$$

TT+118.165×FN-29.515×D+168.613

where *CI*_*p*_*hi*_ represents the piano-harmonic interval consonance index; *CI*_*p*_*mi*_ represents the piano-melodic interval consonance.

##### Visual perception predication model

This section mainly introduced the construction of the visual perception predication model (Input: the interval consonance attribute; Output: one multi-color perceptual attribute) by the linear algorithm.

**FIGURE 6:**
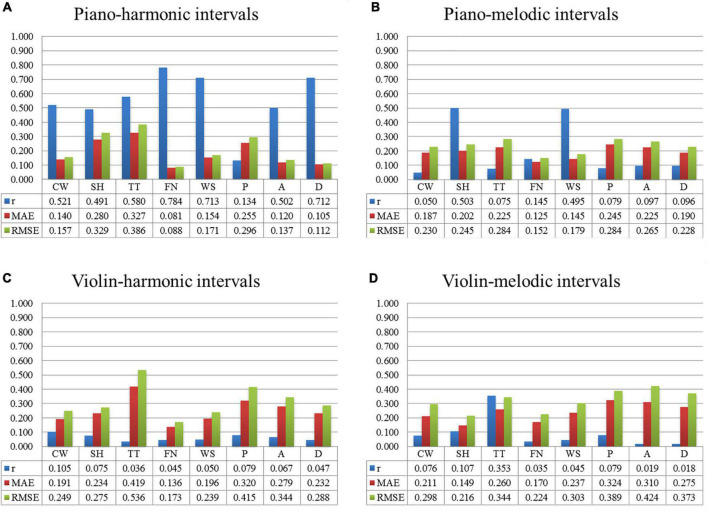
The prediction results of each multi-color perceptual attribute by the interval consonance index. **(A)** The predication results by piano-harmonic intervals. **(B)** The predication results by piano-melodic intervals. **(C)** The predication results by violin-harmonic intervals. **(D)** The predication results by violin-melodic intervals.

Figure 6 shows the prediction results of each multi-color perceptual attribute by the interval consonance index. Among them, Figure 6A shows the prediction results with the piano-harmonic interval consonance index as the input, Figure 6B shows the prediction results with the piano-melodic interval consonance index as the input, Figure 6C shows the prediction results with the violin-harmonic interval consonance index as the input, and Figure 6D shows the prediction results with the violin-melodic interval consonance index as the input.

It can be seen that, when the input was the consonance index of the piano-harmonic interval, the predication performance of each multi-color perceptual attribute, namely “cool/warm,” “transparent/turbid,” “far/near,” “weak/strong,” arousal, and dominance, is good. When the input was the consonance index of the piano-melodic interval, both the “soft/hard” multi-color perceptual attribute and the “weak/strong” multi-color perceptual attributes are well predicted. When the input was the consonance index of the intervals played by violin, the prediction results are bad. The satisfactory models predicted by the consonance index of piano intervals are shown in Equation (17)–(23).


(17) $$C\,W=-0.008\times C\,I_{p\,\_\,h\,i}+3.175$$

(18) $$T\,T=-0.023\times C\,I_{p\,\_\,h\,i}+3.506$$

(19) $$F\,N=-0.008\times C\,I_{p\,\_\,h\,i}+3.364$$

(20) $$W\,S=-0.013\times C\,I_{p\,\_\,h\,i}+3.380$$

(21) $$A=-0.007\times C\,I_{p\,\_\,h\,i}+2.958$$

(22) $$D=-0.009\times C\,I_{p\,\_\,h\,i}+2.264$$

(23) $$S\,H=-0.012\times C\,I_{p\,\_\,m\,i}+3.272$$

where *CI*_*p*_*hi*_ represents the consonance index of the piano-harmonic interval; *CI*_*p*_*mi*_ represents the consonance index of the piano-melodic interval.

Among the audio-visual cross-modal matching models between the multi-color perceptual attributes and the interval consonance attribute constructed by the MLR algorithm, the prediction results of some attributes are relatively better, especially the prediction models between the piano-harmonic interval consonance index and the multi-color perceptual attributes, which are better than the other three types of intervals. In addition, the accuracy of the multi-color perceptual attributes predicted by the interval consonance index is better than that of the interval consonance index predicted by the multi-color perceptual attributes, indicating that the one-way synesthesia channel from multiple colors to combined tones is more easily. In addition, the predication accuracy of the audio perception models is better than that of the visual perception models. Therefore, more audio perceptual attributes need to be extracted as the input besides the interval consonance attribute.

#### Non-linear model construction

In section “Linear model construction,” some audio-visual cross-modal matching models constructed by the MLR algorithm have low accuracy, and the possible reason is that there is not a simple linear relationship between some audio-visual perceptual attributes. Therefore, this article adopted the classical non-linear machine learning algorithms, namely the Support Vector Regression (SVR) algorithm (Drucker et al., 1996), the Random Forest (RF) algorithm (Breiman, 2001), and the Back Propagation (BP) neural network algorithm (Goodfellow et al., 2016) to further explore the non-linear audio–visual relationship.

##### Audio perception predication model

This section mainly introduced the construction of the audio perception predication model (Input: the multi-color perceptual attributes; Output: the interval consonance attribute) by the non-linear algorithms.

The weka software (version 3.8.3; The University of Waikato, Hamilton, New Zealand) with functions of machine learning and data mining was adopted to construct the model. For the SVR algorithm, the KBF kernel *K*(*x*,*y*) = *e*^(−*gamma*(*x*,*y*)^2^)^ with the original value *gamma* = 0.01 was selected. For the RF algorithm, the number of the decision trees was randomly sampled by the Bootstrap algorithm in the bagging strategy. For the BP neural network algorithm, there were three layers in total with one hidden layer, and the hidden layer contained four nodes. For hyper-parameters optimizing, the steps were divided into two steps. Firstly, the randomly selection was used to realize the random matching and selection of the hyper-parameters. Then, based on the optimal result of the random matching, several values in the adjacent range were selected, and the final value of the hyper-parameter was determined by the grid search method.

The Pearson’s correlation coefficient (*r*) were used to evaluate the prediction accuracy of these three machine learning algorithms. In addition, the *r* value of each MLR model was listed together, so as to further study whether the relationship between audio-visual perceptual attributes is linear or non-linear. Table 7 shows the comparison on the modeling results of the interval consonance index predicted by the multi-color perceptual attributes.

**TABLE 7:** The comparison on the modeling results of the interval consonance index predicted by the multi-color perceptual attributes.

	The Pearson’s correlation coefficient			
	
	MLR	SVR	RF	BP
Piano-harmonic interval	0.745	**0.783**	0.650	0.254
Piano-melodic interval	**0.546**	0.216	0.211	0.214
Violin-harmonic interval	0.115	0.103	0.045	**0.134**
Violin-melodic interval	0.081	0.070	**0.133**	0.102

The algorithm which has the best prediction result of the four types of intervals is highlighted in bold respectively.

It can be seen that, except for the BP neural network algorithm, the other three algorithms are all feasible to predict the piano-harmonic interval consonance index, indicating that the multi-color perceptual attributes quantified in this article are well correlated with the piano-harmonic interval attribute. And the SVR algorithm has the best performance on piano-harmonic interval consonance index predicting, indicating the non-linear relationship. The MLR algorithm has the best performance on piano-melodic interval consonance index predicting, indicating the linear relationship. However, the four algorithms are bad at predicting the consonance index of the violin intervals, indicating that there is no correlation between the consonance attribute of the violin intervals and the multi-color perceptual attributes.

##### Visual perception predication model

This section mainly introduced the construction of the visual perception predication model (Input: the interval consonance attribute; Output: one multi-color perceptual attribute) by the non-linear algorithms.

**FIGURE 7:**
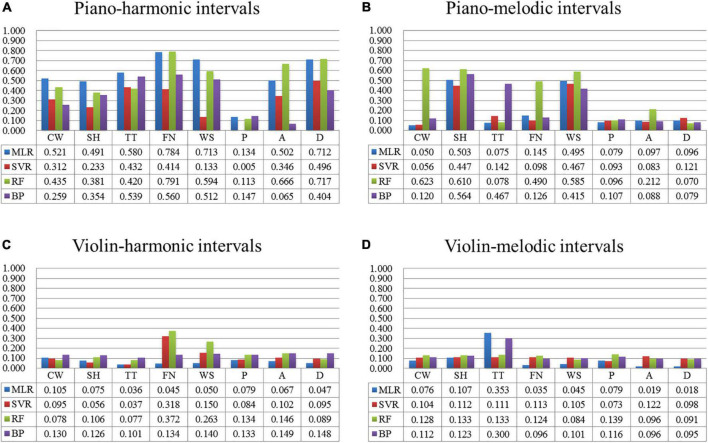
The comparison on the modeling results of four algorithms of each multi-color perceptual attribute by the interval consonance index. **(A)** The comparison of four algorithms predicated by piano-harmonic intervals. **(B)** The comparison of four algorithms predicated by piano-melodic intervals. **(C)** The comparison of four algorithms predicated by violin-harmonic intervals. **(D)** The comparison of four algorithms predicated by violin-melodic intervals.

Figure 7 shows the comparison on the modeling results of four algorithms for predicting the multi-color perceptual attributes by the interval consonance index. Among them, Figure 7A shows the prediction results with the piano-harmonic intervals as the input, Figure 7B shows the prediction results with the piano-melodic intervals as the input, Figure 7C shows the prediction results with the violin-harmonic intervals as the input, and Figure 7D shows the prediction results with the violin-melodic intervals as the input.

The conclusions are below. (1) When the input was the consonance index of the piano-harmonic interval, the MLR prediction results (*r* = 0.5) for the multi-color perceptual attributes, namely “cool/warm,” “transparent/turbid,” and “weak/strong” are better. And the RF algorithm plays good at predicting “far/near,” arousal, and dominance, indicating that these three multi-color perceptual attributes have the non-linear relationship with the interval consonance attribute. (2) When the input was the consonance index of the piano-melodic interval, the RF prediction results (*r* = 0.5) for the multi-color perceptual attributes, namely “cool/warm,” “soft/hard,” and “weak/strong” are better, indicating the non-linear relationship. (3) However, similar to the MLR algorithm, other three non-linear machine learning algorithms did not perform well in predicating the consonance index of violin intervals, indicating that there is little correlation between the violin-interval consonance attribute and the multi-color perceptual attributes.

Through the comparison on the modeling results of the four algorithms, the following conclusions are obtained. (1) It is further confirmed that there is a strong correlation between some multi-color perceptual attributes and the interval consonance attribute, especially the piano-harmonic interval attribute. (2) It is feasible to use the machine learning algorithms to construct the prediction model of the piano-harmonic interval consonance attribute by the multi-color perceptual attributes, which plays a good prediction performance. (3) It is feasible to construct the linear prediction models of some multi-color perceptual attributes (namely “cool/warm,” “soft/hard,” “transparent/turbid,” “far/near,” “weak/strong,” arousal, and dominance) by the consonance index of the piano-harmonic interval. (4) It is feasible to construct the non-linear prediction models of some multi-color perceptual attributes (namely “cool/warm,” “soft/hard,” and “weak/strong”) by the consonance index of the piano-melodic interval. (5) However, other prediction models will not perform well, especially when the input is a sustainable sound signal (e.g., violin).

## Conclusion

This article focused on the association relationship between the perceptual attributes of multiple colors and combined tones, analyzed the correlation between the audio-visual perceptual attributes, and finally constructed the audio-visual cross-modal matching models. The main contributions are below. (1) Construct the multi-color material set and the interval material set, and quantify the multi-color perceptual attributes and interval consonance attribute. (2) Design and implement the audio-visual cross-modal matching subjective evaluation experiment. (3) Analyze the correlation between the perceptual attributes of multiple colors and intervals, and prove that there is a correlation between audio-visual perceptual attributes. (4) Construct the audio-visual cross-modal matching model between audio-visual perceptual attributes, and further study the relationship is linear or non-linear.

The research results of this article have basically achieved the expectation, but there is still room for improvement and further research. Specifically, the future work can be carried out in the following directions. (1) Extract the low-level objective physical parameters which are suitable for perceptual description to realize the construction of the matching model without human participation. (2) To further study the basic composition principles of music and paintings, and construct the audio-visual cross-modal matching models, so as to provide more data support for practical applications.

## Data availability statement

The original contributions presented in this study are included in the article/Supplementary material, further inquiries can be directed to the corresponding author.

## Ethics statement

Ethical review and approval was not required for the study on human participants in accordance with the local legislation and institutional requirements. The patients/participants provided their written informed consent to participate in this study.

## Author contributions

SW and JL designed the whole research and the audio-visual cross-modal matching subjective evaluation experiment. JL and JJ constructed the material set. SW quantified the multi-color perceptual attributes. JL quantified the interval consonance attribute. XL and QH implemented the subjective matching experiment, carried out the correlation analysis, and constructed the audio-visual cross-modal matching model. XL and SW drafted the manuscript. JZ revised the manuscript and supervised the whole process of the research. All authors edited and approved the manuscript.
