# Supplementary material for: Cross-modal association analysis and matching model construction of perceptual attributes of multiple colors and combined tones
Source: Front Psychol. 2022 Dec 6;13:970219. doi: 10.3389/fpsyg.2022.970219 (PMC9763593; doi:10.3389/fpsyg.2022.970219)
Supplement: Supplementary file 1 [file Data_Sheet_1.docx]

# Appendix

**TABLE A1** The 50 multi-color materials with their image categories

| Category | Image descriptive word | Multi-color material | Number |
| --- | --- | --- | --- |
| 1 | Lovely | 1, 6 | 2 |
| 2 | Romantic | 3, 4, 5 | 3 |
| 3 | Refreshing | 15, 20 | 2 |
| 4 | Nature | 2, 9, 10, 12, 17, 19, 22 | 7 |
| 5 | Leisurely | 11, 16, 21, 26, 31 | 5 |
| 6 | Elegant | 7, 8, 13, 14 | 4 |
| 7 | Exquisite | 18, 24, 29, 34 | 4 |
| 8 | Cool/relaxed | 25, 30, 35 | 3 |
| 9 | Dynamic | 36, 41, 46 | 3 |
| 10 | Luxurious | 27, 32, 37 | 3 |
| 11 | Classic | 28, 33 | 2 |
| 12 | Sophisticated | 23, 38, 50 | 3 |
| 13 | Modern | 39, 40, 45, 49 | 4 |
| 14 | Rough | 42 | 1 |
| 15 | Classic/sophisticated | 43, 44, 47 | 3 |
| 16 | Formal | 48 | 1 |

**TABLE A2** The 50 multi-color materials with their values of *L**, *a**, and *b** in the CIELAB color space

| No | Material | $\boldsymbol{L}_{\boldsymbol{1}}^{\boldsymbol{*}}\boldsymbol{a}_{\boldsymbol{1}}^{\boldsymbol{*}}\boldsymbol{b}_{\boldsymbol{1}}^{\boldsymbol{*}}$ | $\boldsymbol{L}_{\boldsymbol{2}}^{\boldsymbol{*}}\boldsymbol{a}_{\boldsymbol{2}}^{\boldsymbol{*}}\boldsymbol{b}_{\boldsymbol{2}}^{\boldsymbol{*}}$ | $\boldsymbol{L}_{\boldsymbol{3}}^{\boldsymbol{*}}\boldsymbol{a}_{\boldsymbol{3}}^{\boldsymbol{*}}\boldsymbol{b}_{\boldsymbol{3}}^{\boldsymbol{*}}$ | No | Material | $\boldsymbol{L}_{\boldsymbol{1}}^{\boldsymbol{*}}\boldsymbol{a}_{\boldsymbol{1}}^{\boldsymbol{*}}\boldsymbol{b}_{\boldsymbol{1}}^{\boldsymbol{*}}$ | $\boldsymbol{L}_{\boldsymbol{2}}^{\boldsymbol{*}}\boldsymbol{a}_{\boldsymbol{2}}^{\boldsymbol{*}}\boldsymbol{b}_{\boldsymbol{2}}^{\boldsymbol{*}}$ | $\boldsymbol{L}_{\boldsymbol{3}}^{\boldsymbol{*}}\boldsymbol{a}_{\boldsymbol{3}}^{\boldsymbol{*}}\boldsymbol{b}_{\boldsymbol{3}}^{\boldsymbol{*}}$ |
| --- | --- | --- | --- | --- | --- | --- | --- | --- | --- |
| 1 | 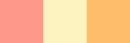 | 74/38/25 | 96/-2/26 | 82/18/50 | 26 | 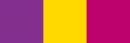 | 36/46/-36 | 88/2/87 | 42/68/-5 |
| 2 | 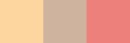 | 88/9/33 | 75/8/15 | 66/42/23 | 27 | 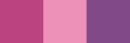 | 47/53/-8 | 71/39/-5 | 40/32/-25 |
| 3 | 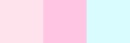 | 93/11/-1 | 85/25/-7 | 97/-11/-5 | 28 | 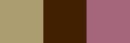 | 65/0/26 | 17/14/24 | 51/28/0 |
| 4 | 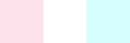 | 92/11/-1 | 100/0/0 | 97/-13/-5 | 29 | 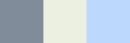 | 58/-2/-9 | 94/-3/7 | 85/-4/-21 |
| 5 | 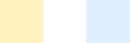 | 96/-1/27 | 99/0/0 | 94/-2/-10 | 30 | 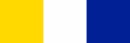 | 88/2/87 | 99/0/0 | 19/32/-67 |
| 6 | 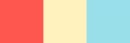 | 61/64/41 | 96/-1/27 | 85/-21/-13 | 31 | 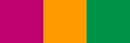 | 42/68/-5 | 74/32/78 | 54/-48/31 |
| 7 | 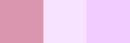 | 70/29/-2 | 92/11/-11 | 87/20/-20 | 32 | 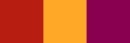 | 40/59/49 | 76/27/73 | 30/54/-5 |
| 8 | 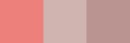 | 66/42/23 | 76/10/6 | 65/14/7 | 33 | 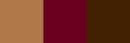 | 56/18/34 | 21/43/15 | 17/14/24 |
| 9 | 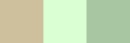 | 78/1/20 | 97/-18/17 | 77/-16/15 | 34 | 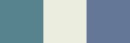 | 52/-13/-11 | 93/-2/7 | 49/-1/-20 |
| 10 | 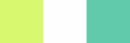 | 93/-25/61 | 99/0/0 | 74/-38/6 | 35 | 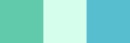 | 74/-38/6 | 96/-16/4 | 71/-27/-18 |
| 11 | 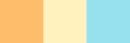 | 82/18/50 | 96/-1/27 | 85/-21/-14 | 36 | 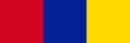 | 44/69/46 | 20/32/-67 | 88/2/87 |
| 12 | 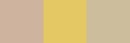 | 75/8/15 | 82/1/53 | 77/2/19 | 37 | 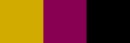 | 72/5/74 | 29/54/-7 | 0/0/0 |
| 13 | 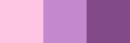 | 86/24/-6 | 65/32/-27 | 40/32/-25 | 38 | 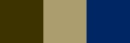 | 22/0/30 | 65/0/27 | 16/0/-41 |
| 14 | 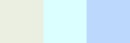 | 94/-3/6 | 97/-12/-4 | 85/-4/-21 | 39 | 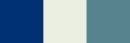 | 21/9/-43 | 94/-3/6 | 52/-13/-11 |
| 15 | 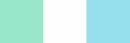 | 86/-30/6 | 99/0/0 | 85/-21/-14 | 40 | 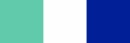 | 74/-38/6 | 99/0/0 | 20/33/-68 |
| 16 | 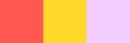 | 61/64/41 | 88/2/81 | 87/20/-20 | 41 | 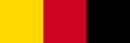 | 88/2/87 | 44/69/46 | 0/0/0 |
| 17 | 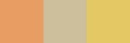 | 71/25/42 | 78/1/20 | 82/1/53 | 42 | 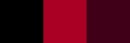 | 0/0/0 | 36/60/34 | 11/31/4 |
| 18 | 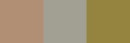 | 62/10/20 | 66/-1/7 | 56/0/39 | 43 | 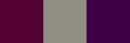 | 16/38/-6 | 59/-2/6 | 12/35/-27 |
| 19 | 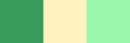 | 57/-41/25 | 96/-1/27 | 90/-40/26 | 44 | 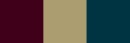 | 11/31/3 | 65/0/26 | 19/-12/-14 |
| 20 | 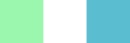 | 90/-40/26 | 100/0/0 | 71/-26/-19 | 45 | 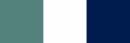 | 51/-18/-2 | 99/0/0 | 11/8/-34 |
| 21 | 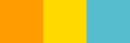 | 74/32/78 | 88/2/87 | 71/-27/-18 | 46 | 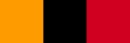 | 73/32/78 | 0/0/0 | 44/70/47 |
| 22 | 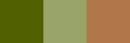 | 38/-15/44 | 65/-11/30 | 56/19/35 | 47 | 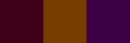 | 11/31/4 | 33/24/43 | 12/35/-27 |
| 23 | 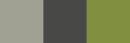 | 66/-2/7 | 31/0/1 | 57/-14/39 | 48 | 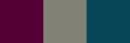 | 16/38/-7 | 54/-2/7 | 27/-14/-16 |
| 24 | 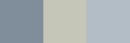 | 58/-3/-9 | 80/-2/7 | 76/-2/-6 | 49 | 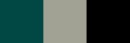 | 27/-21/-4 | 66/-2/7 | 0/0/0 |
| 25 | 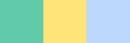 | 74/-38/6 | 91/0/55 | 85/-4/-21 | 50 | 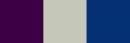 | 12/35/-27 | 80/-3/7 | 21/10/-44 |

*Note: 1 repersents the left color, 2 represents the middle color, and 3 represents the right color.*
